# Sensillar Ultrastructure of the Antennae and Maxillary Palps of the Warble Fly *Oestromyia leporina* (Pallas, 1778) (Diptera: Oestridae)

**DOI:** 10.3390/insects15080574

**Published:** 2024-07-28

**Authors:** Zhuowei An, Xinyu Li, Qike Wang, Wentian Xu, Dong Zhang

**Affiliations:** 1 School of Ecology and Nature Conservation, Beijing Forestry University, Qinghua East Road 35, Beijing 100083, China; anlinsener@163.com (Z.A.); xuwt720@bjfu.edu.cn (W.X.); 2 The College of Forestry, Beijing Forestry University, Qinghua East Road 35, Beijing 100083, China; lixinyubjfu@bjfu.edu.cn; 3 School of BioSciences, University of Melbourne, Melbourne, VIC 3010, Australia; wang.q@unimelb.edu.au

**Keywords:** *Oestromyia leporina*, antenna, maxillary palps, sensilla, sensory pits, morphology, ultrastructure

## Abstract

**Simple Summary:**

Few synapomorphy is available in the important parasitic family Oestridae to clarify its phylogenetic relationships. We used scanning electron microscopy to investigate the morphological structure and ultrastructure of antennae and maxillary palps of male *Oestromyia leporina*, describing the sensillar morphology, types, and distribution. Interestingly, this species has the most complex maxillary palps that are known in Calyptratae. We then proposed that the possible synapomorphy in features of antennal arista by comparing between all published Oestridae species. This study indicates the value of the morphology of antennae and maxillary palps in taxonomy studies in Oestridae and calls for more in-depth studies.

**Abstract:**

Despite the development of molecular techniques, morphological phylogeny still remains integral in underpinning the relationship between some clades of Calyptratae, especially the ones with fast radiation, such as those in Oestridae (Diptera: Brachycera), yet few synapomorphy has been proposed for adults in this family. Using scanning electron microscopy, we investigated the morphological structure and ultrastructure of the antennae and maxillary palps of adult *Oestromyia leporina* (Hypodermatinae, Oestridae). One type of trichoid sensillum (Tr), three types of basiconic sensilla (Ba I, Ba II, and Ba III), one type of coeloconic sensillum (Co I), and one type of clavate sensillum (Cl) were found on the antennal postpedicel. Surprisingly, this species has the most complex types of sensilla on the maxillary palps that have been reported in Calyptratae so far, with two types of coeloconic sensilla (Co II and Co III) and two types of mechanoreceptors. We then identified three common characteristics on the arista of Oestridae (Hypodermatinae, Oestrinae, Gasterophilinae and Cuterebrinae) that are potential synapomorphies. These characteristics indicate the value of the morphology of maxillary palps and aristae in taxonomy studies of Calyptratae.

## 1. Introduction

With the burgeoning development of molecular techniques, the accumulation of molecular data has enabled the reconstruction of much of the evolutionary tree of Calyptratae (Diptera: Brachycera) and resolved some long-lasting debates in clades such as Muscidae, Calliphoridae, and Gasterophilinae [1,2,3,4]. However, morphology, especially ultrastructural studies, remain integral in underpinning the phylogenetic relationship of some clades of Calyptratae. Morphological characteristics are powerful independent sources for testing the accuracy of phylogenetic trees and discovering new branches [5], and understanding how different species adapt to the environment by shaping their structure, biology, and behavior [6]. This information is especially valuable for lineages with fast radiative adaptation when molecular phylogeny retrieves an unstable tree structure, such as in the Oestridae [7].

Few synapomorphies have been proposed in adult Oestridae so far. Current synapomorphies of this family are based on larvae, including the strong mouth hooks (in Gasterophilidae), the complete coverage of the body by spines (in Cuterebrinae), and the larger fronto-orbital tubercles compared to *Gedoelstia* and *Oestrus* (in *Rhinoestrus*) [7]. This is possibly due to the difficulty of accumulating sufficient adult specimens for this relatively rare parasitic family whose history is largely unknown, especially for Hypodermatinae and Sarcophorinae [7]. The lack of synapomorphies in adult Oestridae makes it challenging to identify collected specimens and to validate the taxonomic status of this clade.

The morphology of antennae and maxillary palps could clarify taxonomic and phylogenetic relationships in Calyptratae [8,9]. Zhang et al. (2016) reconstructed the phylogenetic tree of Gasterophilinae using antennal characteristics, which corroborates the tree established with comprehensive molecular data [4,8]. Xu et al. (2022a) proposed that the bottle-shaped sensilla in the antennal sensory pits constituted a synapomorphy of Sarcophagidae, which adds an identification feature to this family, after examining the antennal ultrastructure of all of its three subfamilies [9]. Antennae and maxillary palps are the main olfactory and gustatory organs of insects and play an indispensable role in the search for food, hosts, oviposition sites, and mates [10]; thus, these structures have been subjected to high selection pressure, resulting in a high level of morphological and functional diversity [11,12,13].

*Oestromyia leporina* (Pallas, 1778) is a rare obligate hypodermic parasitic fly that belongs to the subfamily Hypodermatinae of Oestridae [14]. The larvae of *O. leporina* inhabit the skin of Muridae and Leporidae after hatching but do not migrate subcutaneously after invasion [15,16]. Adult *O. leporina* are diurnal, commonly found near the caves of rodents, and inactive for most of the day [17]. This species is generally considered univoltine, adults mostly occur for about one month per year [14]. Examining the antennal and maxillary palp characters of *O. leporine* makes important addition to the scarce understanding of this subfamily and may lead to the discovery of additional synapomorphy for adult Oestridae. Despite the records of other clades of Oestridae, among the eleven genera of Hypodermatinae [7], the antennal structure of only *Hypoderma* and *Portschinskia* has been described in detail so far [18,19].

This study used scanning electron microscopy to investigate the morphological structure of antennae and maxillary palps and the ultrastructure of sensilla in *O. leporina*. We provided a comprehensive description of the morphological structure of antennae and maxillary palps in adult *Oestromyia* species and recorded their types and distribution. We then summarized the findings of this paper and the characteristics described in previous studies to identify possible synapomorphies of Oestridae.

## 2. Materials and Methods

### 2.1. Acquisition of Samples

Specimens were collected in Haibei Prefecture, Qinghai Province, China, in July 2011. Due to the difficulty of collecting this species, three adult male *O. leporina* were examined in this study. All specimens were pinned as museum samples and air dried on site.

### 2.2. Morphological Analyses under Scanning Electron Microscopy

The morphology of the antennae was examined with an Olympus SZX16 stereoscopic microscope (Olympus Corp., Tokyo, Japan). The head of each specimen was rehydrated in phosphate-buffered saline (pH 7.4) for 30 min. The antennae were then dissected from the head and cleaned with detergent in a sonicator. To examine the sensilla in the sensory pits, antennae were cut longitudinally with a razor blade. After dehydration using a graded ethanol series (twice 15 min each in 60%, 70%, 80%, 90%, 95%, and 100% ethanol), the antennae were mounted on round aluminum stubs (15 mm radius, 6 mm in height) with double-sided adhesive tape and left in a desiccator for 24 h to dry. Then, samples were spatter coated with gold and observed using a HITACHI S3400 scanning electron microscope (Hitachi Corp., Tokyo, Japan) at the Microscopy Core Facility, Biological Technology Centre, Beijing Forestry University (Beijing, China). Micrographs were taken at various magnifications, showing the antenna and antennal sensilla. The length and basal diameter of antennal sensilla were determined by measuring their relative size against the scale bar.

The terminology and nomenclature used in this study to define and describe the morphology of antennae, maxillary palps, and sensilla on antennae and maxillary palps follow those described by Xu et al. (2022b) [20], Liu et al. (2022) [21], and Nihei et al. (2022) [22].

## 3. Results

### 3.1. General Description of the Antennae and Maxillary Palps in O. leporina

**Figure 1:**
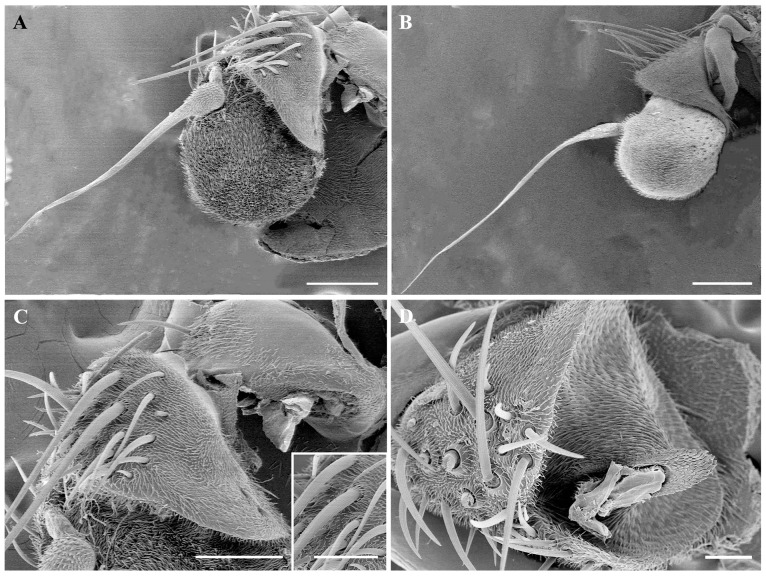
Scanning electron micrographs of general morphology of antenna in *Oestromyia leporina*. (**A**) Dorsolateral and posteroventral surface of antenna. (**B**) Anterodorsal surface of antenna. (**C**) Anterodorsal surface of antennal scape and pedicel, showing the distribution of mechanoreceptors; box shows the longitudinal grooves on mechanoreceptors. (**D**) Morphology of antennal pedicel after removal of the postpedicel. Scale bars: (**A**) 160 μm, (**B**) 200 μm, (**C**) 100 μm, 50 μm in inset, (**D**) 50 μm.

**Figure 2:**
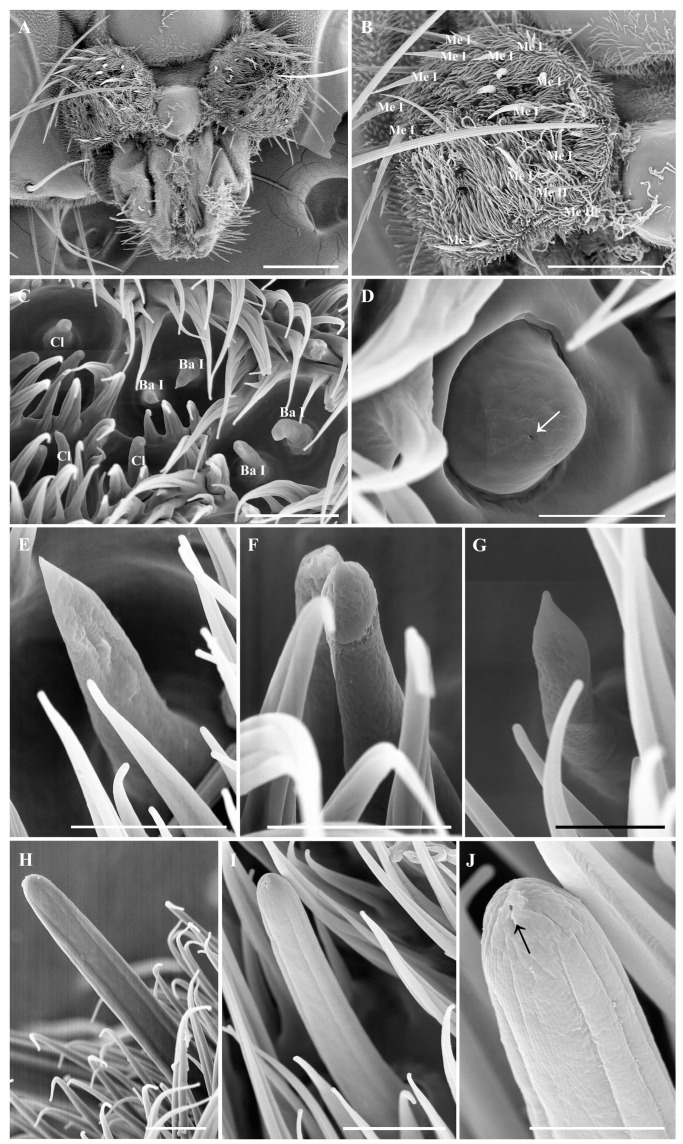
Scanning electron micrographs of general morphology and the sensilla of maxillary palps in *Oestromyia leporina*. (**A**) General view of maxillary palps and beak. (**B**) General view of the right maxillary palp. (**C**) Ba I and Cl in sensory pits on maxillary palp surface. (**D**) Co III, arrow indicates the pore on the sensillar surface. (**E**) General view of Ba I. (**F**,**G**) General view of Cl. (**H**–**J**) Co II, showing it is much longer than Co I; arrow indicates only one pore on the top of the sensilla. Scale bars: (**A**) 150 μm, (**B**) 100 μm, (**C**) 10 μm, (**D**) 3 μm, (**E**) 5 μm, (**F**) 5 μm, (**G**) 2.5 μm, (**H**) 5 μm, (**I**) 5 μm, (**J**) 2 μm. Abbreviations: Me I, mechanoreceptors I; Me II, mechanoreceptors II; Ba I, basiconic sensilla I; Co I, coeloconic sensilla I; Co II, coeloconic sensilla II; Co III, coeloconic sensilla III; Cl, clavate sensilla.

Male *O. leporina* have a pair of oval-shaped antennae in the front of the head between the compound eyes. Each antenna consists of three segments: a scape, a pedicel, and a postpedicel. The long arista grows on the postpedicel (Figure 1A,B). A pair of short, rounded maxillary palps are located on either side of the base of the beak (Figure 2A,B). Although we only observed male *O. leporina*, previous studies of the antennae and maxillary palps of other Calyptratae showed that there is generally no sexual dimorphism in the antennal and maxillary palp structures.

### 3.2. Antennal Scape and Pedicel

The scape is the shortest segment and is attached to the head, with dense hair-like microtrichia and spindly mechanoreceptors at the end part; the base part is bare without microtrichia (Figure 1C). The second segment of the antenna is the pedicel, which is densely covered with microtrichia and is where most of the mechanoreceptors are found. The sharp-tipped mechanoreceptors are relatively straight and strong and arise from protruding sockets. They vary in length, with parallel longitudinal grooves on the cuticular surface (Figure 1C).

### 3.3. Antennal Postpedicel

**Figure 3:**
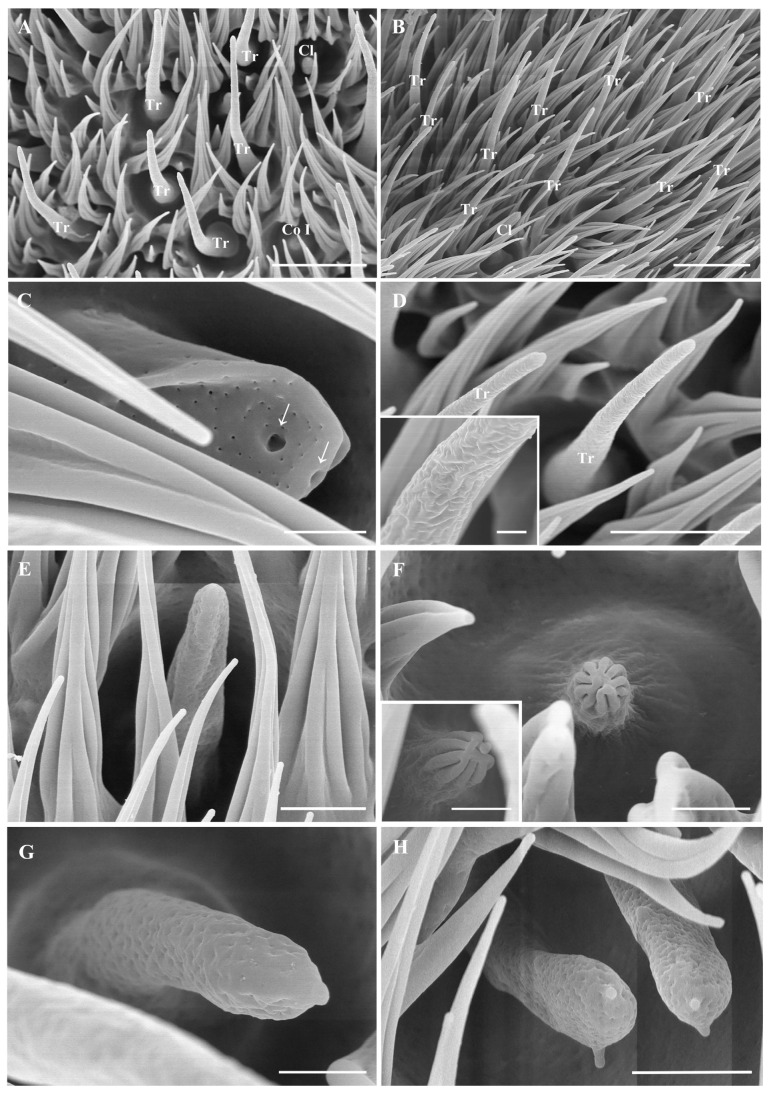
Scanning electron micrographs of general morphology of the sensilla on postpedicel in *Oestromyia leporina*. (**A**,**B**) Distribution of sensilla on the postpedicel surface. (**C**) Ba II, arrow shows two large pores on the end. (**D**) Tr, box shows pores on its surface. (**E**) Ba I, pores can be observed on the cuticular wall. (**F**) Co I, set in shallow depression on postpedicel surface. (**G**) Cl with numerous pores on the cuticular wall. (**H**) Cl with protrusions on the top. Scale bars: (**A**) 10 μm, (B) 10 μm, (**C**) 1 μm, (**D**) 5 μm, 0.5 μm in inset, (**E**) 2.5 μm, (**F**) 1.5 μm, 1 μm in inset, (**G**) 1 μm, (**H**) 2.5 μm. Abbreviations: Tr, trichoid sensilla; Ba I, basiconic sensilla I; Ba II, basiconic sensilla II; Co I, coeloconic sensilla I; Cl, clavate sensilla.

**Figure 4:**
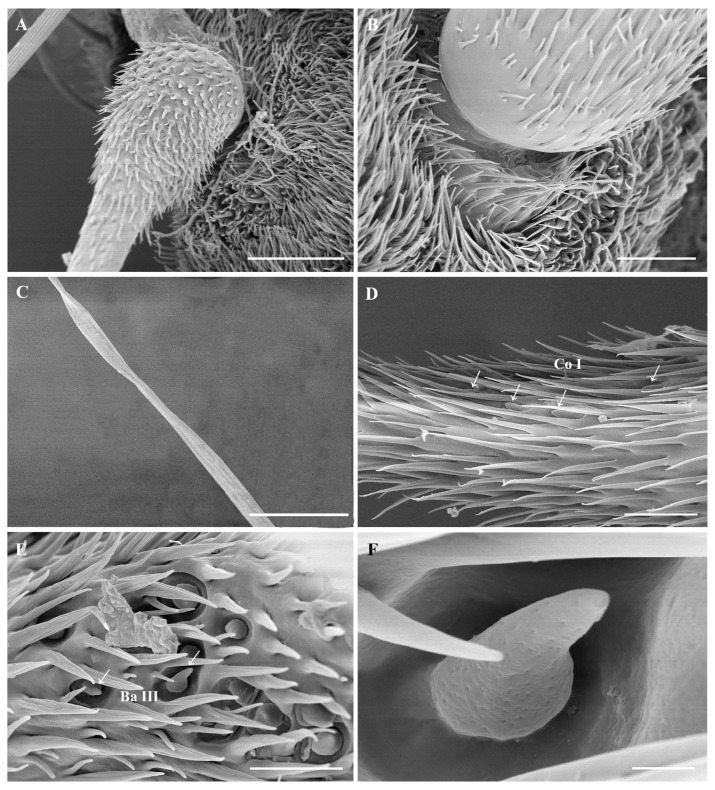
Scanning electron micrographs of the arista in *Oestromyia leporina*. (**A**,**B**) The proximal end of the arista, showing segmented and enlarged base. (**C**) The distal end of the arista, showing the twisted tip. (**D**) The base of the arista, arrows indicate Co I. (**E**) Ba III on the base of the arista, arrows indicate Ba III. (**F**) Enlarged view of Ba III. Scale bars: (**A**) 50 μm, (**B**) 20 μm, (**C**) 50 μm, (**D**) 15 μm, (**E**) 10 μm, (**F**) 1 μm. Abbreviations: Ba III, basiconic sensilla III; Co I, coeloconic sensilla I.

**Figure 5:**
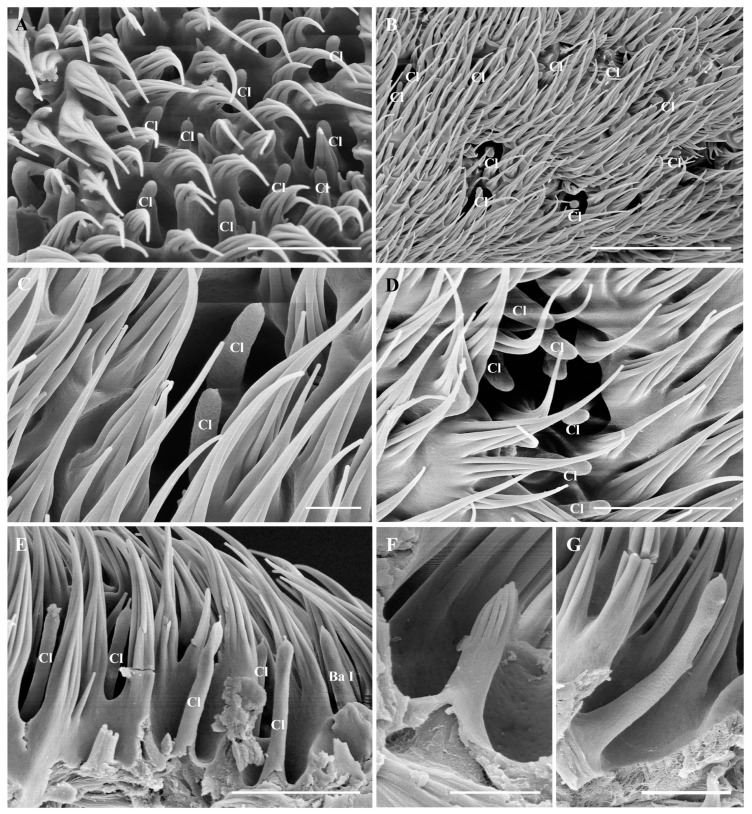
Scanning electron micrographs of sensory pits in *Oestromyia leporina*. (**A**,**B**) Sensory pits on postpedicel surface. (**C**,**D**) Enlarged view of a sensory pit, housing multiple Cl. (**E**–**G**) Lateral view of Ba I, Cl, and Co I in sensory pits. Scale bars: (**A**) 10 μm, (**B**) 30 μm, (**C**) 10 μm, (**D**) 10 μm, (**E**) 10 μm, (**F**) 2.5 μm, (**G**) 5 μm. Abbreviations: Ba I, basiconic sensilla I; Co I, coeloconic sensilla I; Cl, clavate sensilla.

The antennal postpedicel is the longest and most prominent of the three segments; it is oval and can be divided into three surfaces: the anterodorsal surface, the dorsolateral surface, and the posterior ventral surface (Figure 1A,B). The postpedicel is densely covered with hair-like microtrichia and dotted with a large number of sensilla, including trichoid sensilla (Tr) (Figure 3A,B,D), basiconic sensilla (Ba) (Figure 3C,E and Figure 4E,F), coeloconic sensilla (Co) (Figure 3A,F and Figure 4D), and clavate sensilla (Cl) (Figure 3A,B,G,F). In addition, sensory pits housing 1–2 clavate sensilla were found on the postpedicel (Figure 5A,B). The segmented arista with an enlarged base grows on the dorsolateral surface of the postpedicel (Figure 4A,B). The slender distal end of the arista is twisted (Figure 4C). Its surface is densely covered with microtrichia, and coeloconic sensilla I can be observed at the base (Figure 4D).

### 3.4. Maxillary palps

The maxillary palps of *O. leporina* are short, round, and densely covered with short microtrichia (Figure 2B). Numerous sensilla, including mechanoreceptors, basiconic sensilla, clavate sensilla, and coeloconic sensilla, are also present on the maxillary palps (Figure 3C–J).

### 3.5. General Description of the Sensilla on Postpedicel and Maxillary Palps

#### 3.5.1. Trichoid Sensilla

Trichoid sensilla (Tr) are the longest and most numerous of the four types of sensilla, with a length of 18.0 ± 4.2 μm. The base of this sensilla is thick, extending upwards, gradually becoming slender, and ending in a blunt tip. Numerous pores can be observed on its surface (Figure 3D). They are mainly distributed on the dorsolateral surface of the postpedicel.

#### 3.5.2. Basiconic Sensilla

Basiconic sensilla I (Ba I) are shaped like a tapered nail, with a thicker base, a pointed tip, and pores on the surface (Figure 3E). They are 7.9 ± 1.4 μm long and usually shorter than the trichoid sensilla. Basiconic sensilla I are distributed only in the middle and distal end of the postpedicel; their density is lower than that of trichoid sensilla and clavate sensilla. Basiconic sensilla II (Ba II) are 3.9 ± 0.1 μm long, with a nearly cylindrical shape and blunt tips. This sensilla type is densely covered with numerous smaller pores and a few large pores at the tip (Figure 3C). Basiconic sensilla III (Ba III) are found within depressions at the base of the arista. They are tapered and porous, with a length of 3.3 ± 0.2 μm (Figure 4E,F). Basiconic sensilla I are also identified on the maxillary palp surface; they are the same shape as those on the antennal surface but are mainly distributed in sensory pits.

#### 3.5.3. Coeloconic Sensilla

Coeloconic sensilla are the shortest sensilla; there are three types of coeloconic sensilla on *O. leporina.* Coeloconic sensilla I (Co I) are conical, with longitudinal gully grooves on the surface, and the tip is clustered into a finger-like structure, which is located in the postpedicel depression and has a length of 4.6 ± 0.2 μm (Figure 3F). Coeloconic sensilla I are evenly distributed on the postpedicel and are sparser than trichoid sensilla and basiconic sensilla. Numerous coeloconic sensilla I are also found at the base of the arista (Figure 3D).

Two types of coeloconic sensilla are found on the maxillary palps. Coeloconic sensilla II (Co II) are 13.8 ± 1.2 μm in length, and the surface has longitudinal grooves that converge at the top, but no pore was observed on the surface (Figure 2J). Coeloconic sensilla II are the shortest sensilla among the four types of sensilla and are straighter than coeloconic sensilla I, which are usually hidden beneath the microtrichia, whereas coeloconic sensilla II protrude above the microtrichia (Figure 2H,I). Coeloconic sensilla III (Co III) are almost dome-shaped, measuring 3.3 ± 0.1 μm long and 4.1 ± 0.1 μm in diameter, and are located within convex peripheral rings. Their surface is smooth and has one pore on the top (Figure 2D).

#### 3.5.4. Clavate Sensilla

Clavate sensilla (Cl) are characterized by a swollen tip and a porous surface. The length of the clavate sensilla is similar to that of basiconic sensilla, which is about 11.8 ± 0.8 μm (Figure 3G). Clavate sensilla exist in the middle and at the base of the postpedicel, and the density of clavate sensilla on the anterodorsal surface is greater than that of those on the dorsolateral surface and the posterior ventral surface. Occasionally, protrusions can be found on the tip of some clavate sensilla (Figure 3H). The morphology of the clavate sensilla on the maxillary palps is the same as the ones on the postpedicel.

#### 3.5.5. Sensory Pits

Sensory pits are single-compartment depressions with few microtrichia on the antennal postpedicel (Figure 5A,B). They are mainly distributed on the middle and proximal parts of the anterodorsal and posterior ventral surfaces of the postpedicel, and their distribution is relatively concentrated (Figure 1A,B). Generally, 1–3 clavate sensilla can be observed within one sensory pit. Sensory pits are also distributed on the maxillary palp surface, each housing 1–2 basiconic sensilla or clavate sensilla (Figure 2A,C).

#### 3.5.6. Mechanoreceptors on Maxillary Palps

**Figure 6:**
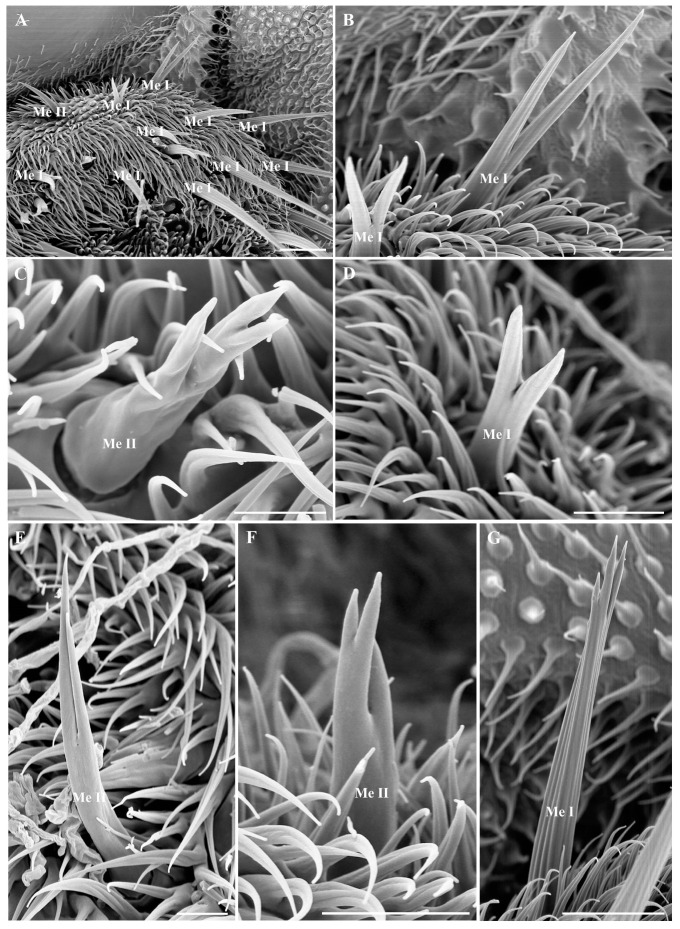
Scanning electron micrographs of mechanoreceptors on the maxillary palps in *Oestromyia leporina*. (**A**) Overview of the mechanoreceptors on maxillary palps. (**B**,**D**) Bifurcated mechanoreceptors with ridges on surface. (**C**) A short trifurcated mechanoreceptor with smooth surface. (**E**) A bifurcated mechanoreceptor with two long branches. (**F**) A trifurcated mechanoreceptor with smooth surface. (**G**) A long trifurcated mechanoreceptor with ridges on the surface. Scale bars: (**A**) 25 μm, (**B**) 10 μm, (**C**) 5 μm, (**D**) 10 μm, (**E**) 10 μm, (**F**) 10 μm, (**G**) 15 μm. Abbreviations: Me I, mechanoreceptors I; Me II, mechanoreceptors II.

Numerous tactile mechanoreceptors with lengths varying from 15.6 to 70.7 µm are identified on the maxillary palps of *O. leporina* (Figure 6A). Based on their surface topography, they can be divided into two types: one with longitudinal ridges and the other with a relatively smooth surface (Figure 6). Interestingly, about half of them are bifurcated or trifurcated (Figure 6B–G). The mechanoreceptors are mostly distributed on the lateral side of the maxillary palps (Figure 2A).

## 4. Discussion

The present study described the ultrastructure of the antennae and maxillary palps in Hypodermatinae using scanning electron microscopy. The morphological structure and distribution of Tr, Ba I, Co I, and Cl on the antennal surface of *O. leporina* are similar to those of other Oestridae species, but two new types of basiconic sensilla (Ba II and Ba III) were found on the antenna postpedicel (Figure 3C and Figure 4F). Two novel coeloconic sensilla (Co II and Co III) (Figure 2D,J) and two types of mechanoreceptors were identified on the maxillary palps (Figure 6). Surprisingly, *O. leporina* has the most complex types of sensilla on the maxillary palps among all the known Calyptratae species.

Similar to other Oestridae species (see Table 1), the slender arista of this species is unbranched, twisted, segmented, and largely smooth, with an enlarged base and coeloconic sensilla I on the surface (Figure 4). We thus compared five microscopic and ultramicroscopic characteristics of the arista that are common in at least some lineages of the four subfamilies of Oestridae (Hypodermatinae, Oestrinae, Gasterophilinae, and Cuterebrinae) with those of other Calyptratae families, including Hippoboscidae, Tachinidae, Sarcophagidae, Muscidae, Fanniidae, Calliphoridae, and Anthomyiidae (Table 1). We identified three common morphological characteristics of the arista of Oestridae, including 1. the twisted tip, 2. the enlarged base, and 3. the surface coeloconic sensilla at the base (except for *Hypoderma lineatum*, *Oestrus ovis*, and *Rhinoestrus purpureus*). These characteristics are common among Oestridae but are absent in other Calyptratae species, and thus, they may be synapomorphies of Oestridae. These qualitative characteristics may be easier to identify in taxonomic studies than the number of sensory pits on the postpedicel, which was proposed by Xu et al. (2022b) [20], although comprehensive comparisons between more species are needed.

The branched sensilla of *O. leporina* is an uncommon feature that has only been reported on the antennae of *Portschinskia magnifica* (Hypodermatinae) and *Rhinoestrus purpureus* (Oestrinae) [23,25]. Branched trichoid sensilla and basiconic sensilla on the antennal surface of *P. magnifica* usually have one long branch or 1–3 small branches (Zhang et al., 2012a, in Figure 3C,D and Figure 4C,D) [23], whereas the branched basiconic sensilla of *R. purpureus* usually have several short branches (Liu et al., 2015, in Figures 2B and 3B) [25]. By comparison, a variety of branched mechanoreceptors on the maxillary palps and branched clavate sensilla with a short spike on the antennae were found in *O. leporina* in this study (Figure 3H and Figure 6). These branched sensilla have been reported in three species of Oestridae so far; thus, they cannot be regarded as a synapomorphy within this family. However, it may not be a coincidence that all the species with branched sensilla are obligatory parasites. The exact function of these branches is unclear; this structure significantly increases the surface area of each sensillum and thus may improve its efficiency in capturing subtle changes in air flow.

Maxillary palps may supplement the function of antennae by providing gustatory and close-range olfactory functions [32,33]. The morphology of Calyptratae maxillary palps generally receives less attention, presumably because of the relatively simple ultrastructural features. For example, one type of basiconic sensillum and one type of mechanoreceptor were identified on the maxillary palps in Muscidae (*Lispe*, *Musca*, and *Hydrotaea*) [20,34,35] and Fanniidae (*Fannia*) [30]. The surprising diversity of sensilla on the maxillary palps of *O. leporina*, including basiconic sensilla I, clavate sensilla I, and coeloconic sensilla II and III (Figure 2), may reflect the importance of maxillary palps to the parasitic lifestyle of this species. These complex sensilla types may provide key morphological characteristics for taxonomic and phylogenetic studies of Oestridae (including Hypodermatinae, Oestrinae, and Cuterebrinae), despite a lack of mouth parts in some lineages (i.e., Gasterophilinae). The morphology of maxillary palps may provide additional synapomorphies for adult flies in Oestridae, although more in-depth morphological comparisons are needed.

## Figures and Tables

**Table 1:** Comparison of morphological characteristics of arista in different families of Calyptratae.

Species	Family	Twisted	Enlarged Base	Segmental	Coeloconic Sensilla at the Base	Branched
*Oestromyia leporina*	Oestridae	Yes	Yes	Yes	Yes	No
*Portschinskia magnifica* [23]	Oestridae	Yes	Yes	Yes	Yes	No
*Hypoderma bovis* [18]	Oestridae	Yes	Yes	Yes	Uncertain	No
*Hypoderma lineatum* [19]	Oestridae	Yes	Yes	Yes	No	No
*Oestrus ovis* [24]	Oestridae	Yes	Yes	Yes	No	No
*Rhinoestrus purpureus* [25]	Oestridae	Yes	Yes	Yes	No	No
*Gyrostigma rhinocerontis* [20]	Oestridae	Yes	Yes	Yes	Yes	No
*Gasterophilus nigricornis* [26]	Oestridae	Yes	Yes	Yes	Yes	No
*Gasterophilus pecorum* [8]	Oestridae	Yes	Yes	Yes	Yes	No
*Gasterophilus nasalis* [8]	Oestridae	Yes	Yes	Yes	Yes	No
*Gasterophilus intestinalis* [8]	Oestridae	Yes	Yes	Yes	Yes	No
*Dermatobia hominis* [27]	Oestridae	Yes	No	Yes	Yes	Yes
*Melophagus ovinus* [28]	Hippoboscidae	No	No	No	No	Yes
*Hippobosca equina* [28]	Hippoboscidae	No	No	No	No	Yes
*Hippobosca longipennis* [28]	Hippoboscidae	No	No	No	No	Yes
*Cylindromyia carinata* [22]	Tachinidae	No	No	Yes	No	No
*Sarcophaga portschinskyi* [9]	Sarcophagidae	No	No	Yes	No	Yes
*Agria mihalyii* [9]	Sarcophagidae	No	No	Yes	No	Yes
*Metopia campestris* [9]	Sarcophagidae	No	No	Yes	No	No
*Lispe longicollis* [21]	Muscidae	No	No	Yes	No	No
*Lispe orientalis* [21]	Muscidae	No	No	Yes	No	No
*Lispe pygmaea* [21]	Muscidae	No	No	Yes	No	No
*Fannia hirticeps* [29]	Fanniidae	No	No	Yes	No	No
*Triceratopyga calliphoroides* [30]	Calliphoridae	No	No	Yes	No	Yes
*Delia platura* [31]	Anthomyiidae	No	No	Yes	No	No

## Data Availability

The data generated by this study are provided here, and they are also available upon request from the corresponding author.
